# Characteristics of interactions at protein segments without non-local intramolecular contacts in the Protein Data Bank

**DOI:** 10.1371/journal.pone.0205052

**Published:** 2018-12-11

**Authors:** Kota Kasahara, Shintaro Minami, Yasunori Aizawa

**Affiliations:** 1 College of Life Sciences, Ritsumeikan University, Noji-higashi, Kusatsu, Shiga, Japan; 2 Exploratory Research Center on Life and Living Systems, National Institutes for Natural Sciences, Myodaiji, Okazaki, Aichi, Japan; 3 School of Life Science and Technology, Tokyo Institute of Technology, Nagatsuda-cho, Midori-ku, Yokohama, Kanagawa, Japan; Russian Academy of Medical Sciences, RUSSIAN FEDERATION

## Abstract

The principle of three-dimensional protein structure formation is a long-standing conundrum in structural biology. A globular domain of a soluble protein is formed by a network of atomic contacts among amino acid residues, but regions without intramolecular non-local contacts are often observed in the protein structure, especially in loop, linker, and peripheral segments with secondary structures. Although these regions can play key roles for protein function as interfaces for intermolecular interactions, their nature remains unclear. Here, we termed protein segments without non-local contacts as *floating* segments and sought them in tens of thousands of entries in the Protein Data Bank. As a result, we found that 0.72% of residues are in floating segments. Regarding secondary structural elements, coil structures are enriched in floating segments, especially for long segments. Interactions with polypeptides and polynucleotides, but not chemical compounds, are enriched in floating segments. The amino acid preferences of floating segments are similar to those of surface residues, with exceptions; the small side chain amino acids, Gly and Ala, are preferred, and some charged side chains, Arg and His, are disfavored for floating segments compared to surface residues. Our comprehensive characterization of floating segments may provide insights into understanding protein sequence-structure-function relationships.

## Introduction

Elucidating the principles of the three-dimensional (3D) structure formation of proteins is a long-standing conundrum in the field of structural biology [1–3]. How a sequence of 20 types of amino acid residues encoded in the standard genetic code (and additional two amino acid residues added via specific translation mechanisms [4]) in a polypeptide determines its 3D structure remains largely unclear. To illumination of this issue, extensive efforts in structural biology have accumulated a massive amount of structural data for proteins in the Protein Data Bank (PDB) [5]. By taking advantage of the wealth of structural data, the field of so-called “structural bioinformatics”, which tackles extracting biological knowledge from structural databases by using techniques of information science, has arisen [6–9]. Statistical analyses of structural elements in the PDB have provided a bird’s eye view on the characterization of the 3D structures of proteins. For example, statistical analyses revealed amino acid propensities associated with several features, e.g., formation of secondary structural elements [10–13] and loop regions [14]. In addition to secondary structure formation, another key feature to establish protein folding is intramolecular contact between amino acid residues distant in the primary structure; in this paper, we refer to this type of intramolecular contact as *non-local* contact, and contacts between neighboring residues are termed *local contacts*. The statistical analyses of the vicinity of amino acid residues in 3D space have been extensively performed to predict and recognize protein folds [15,16]. For example, the residue-wise contact order, defined as the summation over the distance along the sequence between contacting residues, contains significant information regarding 3D structures [17,18]. Propensities of non-local contacts play pivotal roles in establishing folds of globular domains.

However, proteins also have regions without non-local contacts. A typical example is the linker region, which is a flexible segment linking two globular domains. The structural element termed the loop region also tends to have no or only a few non-local contacts. They are also key elements in the 3D structures of proteins. However, in spite of their importance, the nature of the regions without non-local contacts is not well understood.

**Fig 1:**
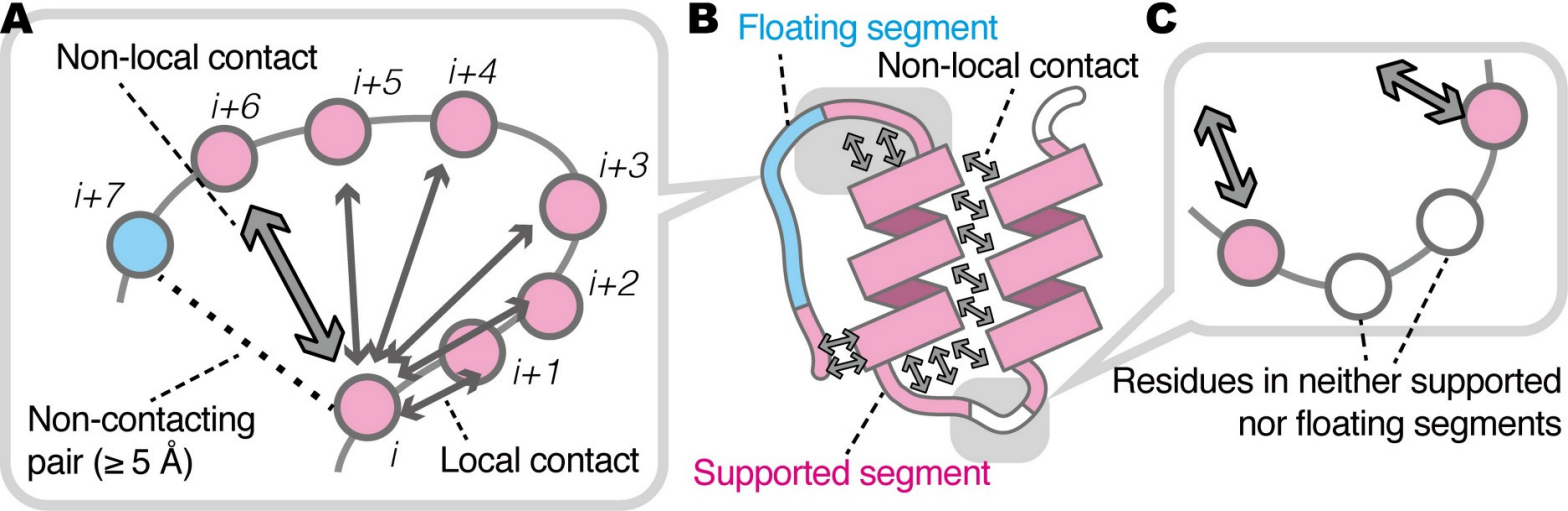
Schematic illustration of *floating* and *supported* segments. (A) Definition of non-local contacts. A circle indicates amino acid residue. When a pair of amino acids interact and are within five residues in the sequence order, this contact is regarded as a local contact (thick gray arrows). When a pair of amino acids interact but are more than five residues apart in the sequence order, this contact is regarded as a non-local contact (the bold arrow). Amino acid pairs further than 5 Å are not considered to be in contact (the dotted line). (B) Definition of floating and supported segments. A unit consisting of more than or equal to three consecutive amino acid residues with and without non-local contacts are defined as floating and supported segments, respectively. (C) Residues that are not in neither in floating nor supported segments. When the neighboring three residues do not have the same properties (white circles), they are not included in the segments.

Here, we performed statistical analyses on PDB entries to investigate the nature of regions without non-local contacts. Tens of thousands of PDB entries were processed, and two types of regions consisting of consecutive amino acid residues were defined: (i) regions with non-local contacts, and (ii) those without non-local contacts. We refer to these regions as (i) *supported* segments and (ii) *floating* segments, respectively (Fig 1). We aim to characterize the floating segments in proteins. On the basis of non-redundant PDB entries, the frequency of these floating segments was analyzed with many features of the segments, e.g., length, secondary structures, accessible surface area (ASA), and intermolecular interactions. We present that a considerable number of residues are floating in protein structures deposited in the PDB. While amino acid preferences of floating segments are similar to those of exposed residues, some amino acids exhibited a unique propensity for floating segments.

## Materials and methods

### Dataset construction

In this study, we constructed two kinds of datasets, named *primary dataset* and *non-redundant datasets*. The latter are subsets of the first. The primary dataset was constructed by extracting entries from a snapshot of the PDB on June 14, 2017, with the following criteria: (i) the entry contains at least one polypeptide, (ii) the number of atoms is less than one million, and (iii) the structure was solved with X-ray crystallography with resolution better than, or equal to, 3.0 Å. Extracting information from the PDB was performed by parsing PDBML [19] with in-house scripts (S1 Data).

Non-redundant datasets were constructed by picking non-redundant entries from the primary dataset. Single-linkage clustering with sequence identity > 40% was performed using the CD-HIT program [20]. In cases where an entry had more than one chain, the sequence identity of the most similar pair of chains was considered. A non-redundant dataset was constructed by random picking of one entry from each cluster. We constructed 100 non-redundant datasets with different random seeds, and thes statistics involved were analyzed.

### Detecting atomic contacts and definition of structural units

In this study, we assessed interatomic contacts between heavy atoms, or atoms other than hydrogen, with the threshold that the interatomic distance is less than 5 Å. When a pair of amino acid residues has at least one interatomic contact, we considered that this pair of residues has an *inter-residue contact*. An inter-residue contact formed between residues of the same molecule is termed an *intramolecular contact*, which can be grouped into two classes: (i) non-local contact for the cases in which the two contacting residues are distant more than five residues in the sequence order (the same threshold has been utilized in the CASP contests [21]), and (ii) local contact for other cases. An *intermolecular contact* is defined as a contact between two different molecules. On the basis of inter-residue contacts, we defined a structural unit named *segment*. A unit consisting of more than three successive amino acid residues with non-local contacts is defined as a *supported* segment and that without non-local contacts is defined as a *floating* segment (Fig 1).

### Analyses

We characterized segments in polypeptide chains by focusing on the following points: (i) segment type, defined as floating or supported, (ii) segment length, (iii) secondary structural elements (SSEs), (iv) accessible surface area (ASA), and (v) types of inter-molecular contact partners. (i) The segment type is signified as *T*_*seg*_ ⊂ {*flo*,*sup*}; *flo* and *sup* mean floating and supported segments, respectively. (ii) Segment length *L*_*seg*_ is the number of consecutive amino acid residues composing the segment. For simplicity, segment lengths fell into three classes: *T*_*len*_ ⊂ {*shoft*,*middle*,*long*}, that were defined as segments with 3–4 amino acids, 5–9 amino acids, and longer than those, respectively. (iii) The type of SSE (*T*_*SSE*_) was assessed by using the DSSP program [22,23]. In this manuscript, we applied three categories of SSE; *T*_*SSE*_ ⊂ {*helix*,*beta*,*coil*}; *helix* was *G*, *H* or *I* in the DSSP classification, *beta* was *B*, *E*, *T*, or *S*, and *coil* was the others. The representative *T*_*SSE*_ of each segment was the most frequent SSE in the residues composing the segment. (iv) The solvent accessibility of each segment was defined as the two levels: surface and buried, *T*_*ASA*_ ⊂ {*sur*,*bur*}. For each amino acid residue, when the ratio of ASA of the residue to that of the same amino acid in Gly-X-Gly motifs is greater than 0.2, the residue is assumed to be surface exposed. Otherwise, it is assumed to be buried. ASA was calculated with the DSSP program [22,23] for the entire structure of each PDB entry (including multimer complexes). (v) Partners of intermolecular contacts fell into three classes: polypeptides, polynucleotides, and chemical compounds. These were defined by the entity type as described in the PDB annotation. We considered non-polymer entities ≥ 300 Da as chemical compounds to eliminate tiny non-specific binders such as sulfuric acids, alcohol, and metallic ions. The type of interaction partner of a segment is denoted as *T*_*int*_ ⊂ {*pep*,*nuc*,*sc*} for polypeptide, polynucleotide, and chemical compound, respectively.

Relative frequencies of various types of segments were analyzed. *F(x)* indicates the relative frequency of segments with the condition *x*,

$$F(x)=\frac{The\;number\;of\;segments\;with\;the\;condition\;x}{The\;total\;number\;of\;segments\;in\;a\;dataset}.$$ (1)

For example, the relative frequency of short segments and that of helix segments are represented as *F(T*_*len*_ *= short)* and *F(T*_*SSE*_ *= helix)*, respectively. For simplicity, they can also be denoted as *F(short)* and *F(helix)*. The conditional relative frequency, the ratio of the number of segments with types *x* and *y* to that with type *y*, is denoted as *F(x|y)*.

In addition, we also analyzed characteristics of residues. The relative frequency of residues with the condition *x* is presented as *F*^*res*^*(x)*. The amino acid type of each residue is denoted as $T_{AA}^{res}\subset \{A,C,D,E,F,G,H,I,K,L,M,N,P,Q,R,S,T,V,W,Y\}$. The propensity score of each amino acid was assessed with the log-odds score.

$$S^{res}(T_{AA}^{res}|x)=\log\left(\frac{F^{res}(T_{AA}^{res}|x)/(1-F^{res}(T_{AA}^{res}|x))}{F^{res}(T_{AA}^{res})/(1-F^{res}(T_{AA}^{res}))}\right),$$ (2)

$$S^{res}(T_{AA}^{res}|x;\;y)=\log\left(\frac{F^{res}(T_{AA}^{res}|x,y)/(1-F^{res}(T_{AA}^{res}|x,y))}{F^{res}(T_{AA}^{res}|y)/(1-F^{res}(T_{AA}^{res}|y))}\right),$$ (3)

where *x* and *y* indicate conditions. We evaluated the amino acid propensities for segment types $S^{res}(T_{AA}^{res}|T_{seg})$, those for interaction partners in each segment type $S^{res}(T_{AA}^{res}|T_{int};T_{seg})$, and those for surface or buried residues $S^{res}(T_{AA}^{res}|T_{ASA})$. For the analysis of the amino acid propensities, consecutive His residues in the C-terminus were eliminated from the statistics, since they should be artificially inserted His-tag sequences.

## Results and discussion

### Database statistics

The primary dataset consisted of 89,038 PDB entries, 115,287 entities, 225,366 chains, and 53,497,598 residues. Single-linkage clustering with 40% sequence identity resulted in 15,351 clusters. Among these, 12,513 clusters (81.51%) had less than, or equal to, five members, and 6,846 clusters (44.60%) of these were singleton clusters (S1 Fig). To estimate the dataset biases, we analyzed 100 non-redundant datasets, each of which was constructed by randomly picking one entry from each cluster. The average and standard deviation (SD) of quantities will be discussed. On average [with SD], a non-redundant dataset consisted of 1.726×10^4^ [37.44] entities, 3.86×10^4^ [140.30] chains, and 8.97×10^6^ [34,917.46] residues.

For the PDB entries, we defined floating segments as having at least three consecutive residues without non-local contacts, and defined supported segments as those with non-local contacts, respectively. The primary dataset included 359,501 floating segments and 4,419,603 supported ones. Out of 53,497,598 residues in the dataset, 25,421,627 residues (47.5%) belonged to either a floating or supported segment (a segment is defined as more than two successive residues with or without non-local contacts; Fig 1C), and the remaining 52.5% of residues that belonged to neither were not analyzed in this study. The average numbers [and SD] of floating and supported segments in the non-redundant datasets were 6.43×10^4^ [387.25] and 7.26×10^5^ [2,960.58], respectively. 0.72% of residues were in floating segments.

### Segment length and secondary structural elements

**Fig 2:**
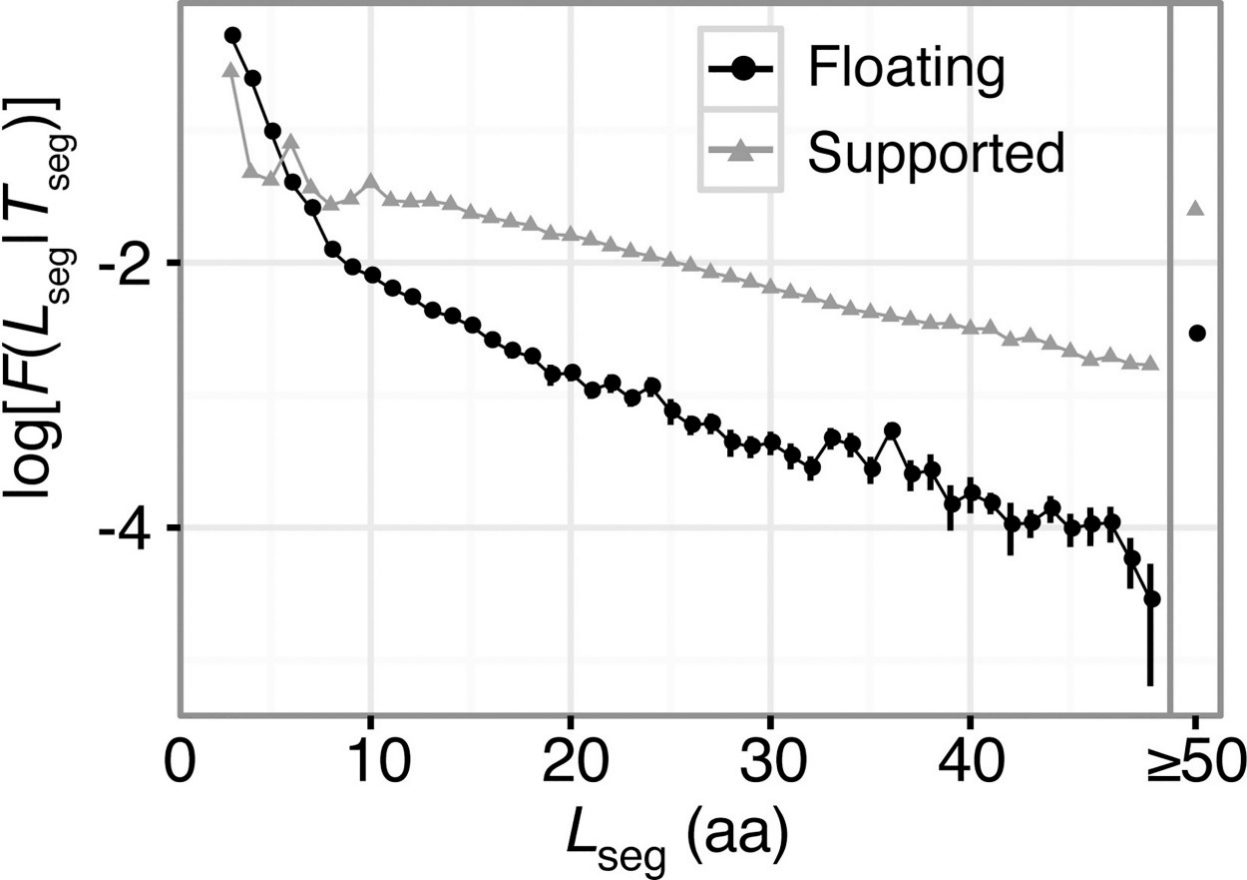
**Histogram of floating (black) and supported (gray) segments regarding segment lengths**. The vertical axis is the log-ratio of the number of segments in each length to that for all length. The right of the plot indicates the values when *L*_seg_ ≥ 50 amino acids. Floating segments tend to consist smaller number of amino acids compared to supported ones. This trend in floating segments inflects around *L*_seg_ = 9 amino acids. Error bars are the standard deviations in 100 non-redundant datasets.

Distributions of the segment length *L*_*seg*_ for each segment type are shown in Fig 2. The number of segments decreases exponentially, along with an increase in the segment length. In particular, the gradient is steep for *L*_*seg*_ < 9 amino acids. A majority of floating segments consist of only three or four residues (*F(short|flo)* = 0.850), and the ratio of floating segments that are longer than nine residues is only *F(long|flo)* = 0.014. However, many supported segments have more than nine residues (*F(long|sup)* = 0.103; *F(short|sup)* = 0.568). Floating segments tend to be shorter than supported ones. This may reflect the fact that longer protein regions without molecular contacts should have higher flexibility. Since it is difficult to determine atomic coordinates of such regions by crystallography, they are not included in this study. The long flexible regions, such as intrinsically disordered regions, are abundant in nature although their atomic coordinates are not recorded in the PDB. Note that in this study we did not consider missing regions in the PDB entries, which means regions without atomic coordinates.

**Fig 3:**
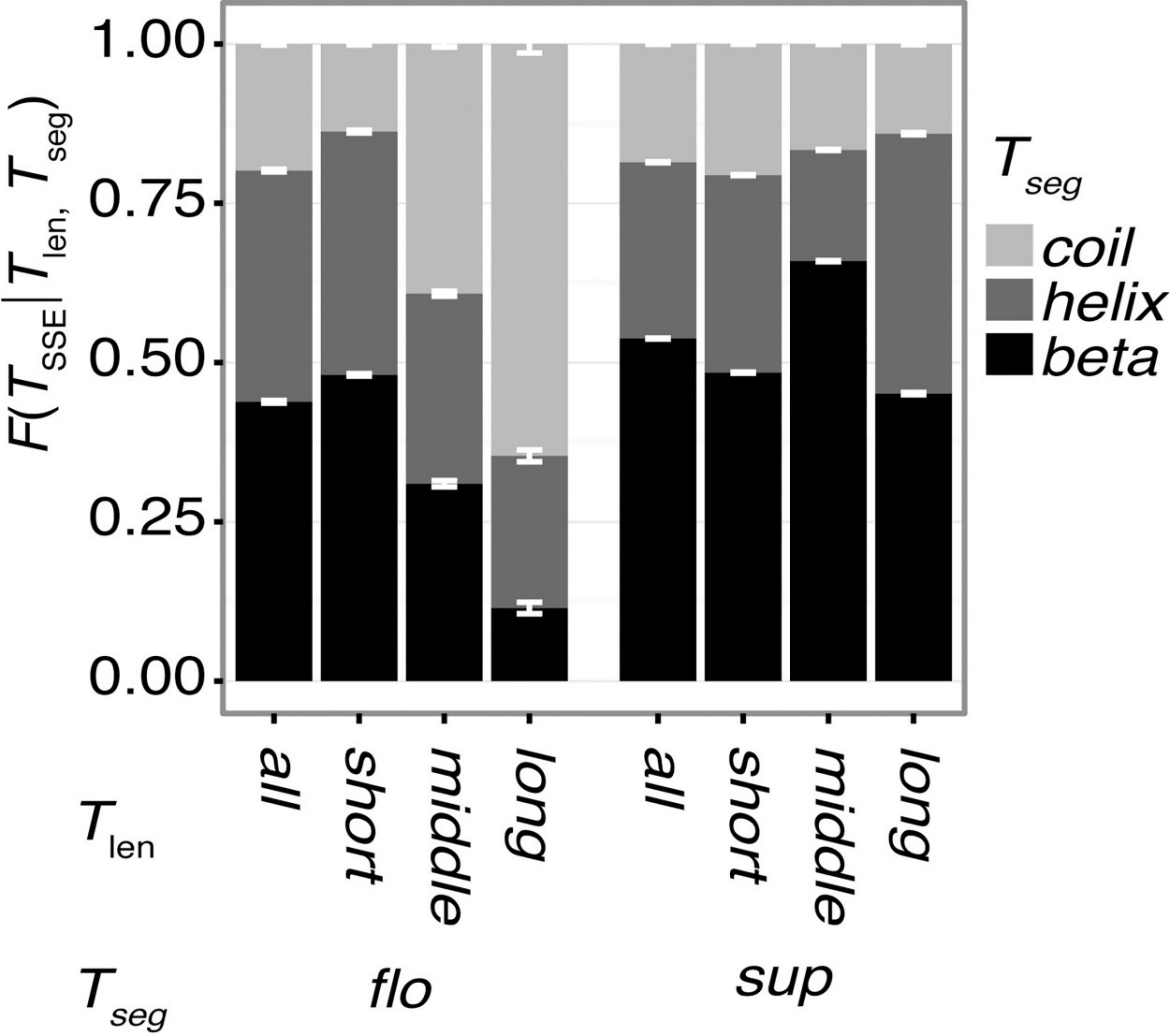
**Ratio of the secondary structural elements (SSE) for each combination of classes:** *T*_len_ and *T*_seg_. This figure indicates that the floating segments tend to disfavor beta structures as the segment length increases, in contrast to supported segments. Black, dark gray, and light gray denote the ratios of *beta*, *helix*, and *coil* structures, respectively. Error bars are the standard deviations in 100 non-redundant datasets.

**Fig 4:**
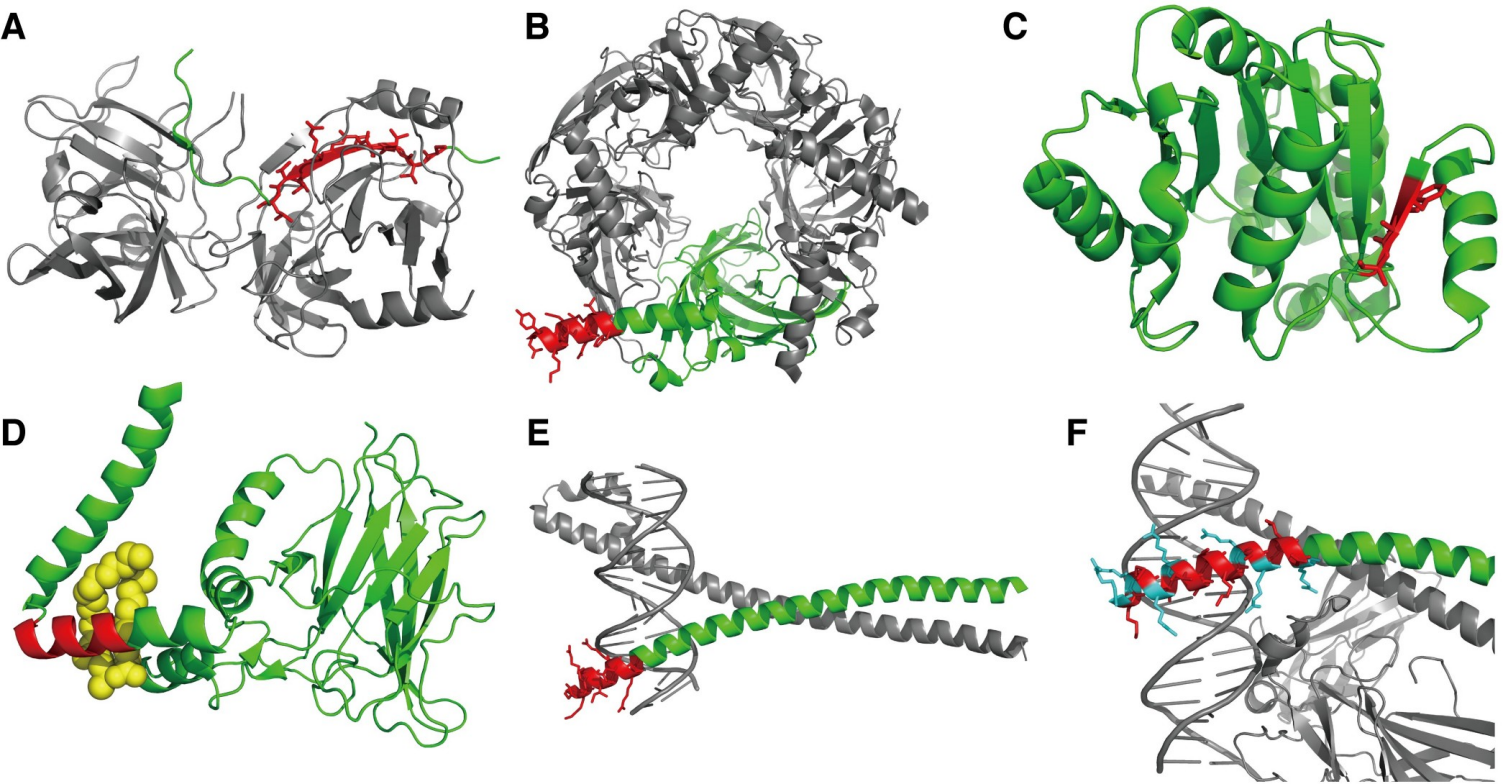
Examples of 3D structures of segments. The target segment, the chain including the segment, and other chains are shown in red, green, and gray, respectively. (A) A long-beta floating segment composing an intermolecular β-sheet (a virus serine protease; PDB ID: 2O8M). (B) A long-helix floating segment at a solvated terminus (an acetylcholine receptor; PDB ID: 2WN9). (C) A supported-short segment in a β-sheet (a putative nucleotide-diphospho-sugar transferase; PDB ID: 3CGX) (D) A floating segment at a chemical compound-binding site. (3-Chlorocatechol 1,2-Dioxygenase; PDB ID: 2BOY). The chemical compound is shown in yellow. (E) A floating-helix segment at a nucleic acid binding site (a bZIP heterodimeric complex; PDB ID: 2WT7). (F) A floating segment with many Arg residues (Jun-Fos heterodimer; PDB ID: 1A02). This segment recognizes the double-stranded DNA.

Segment length relates to the secondary structure formation of segments. Longer segments tend to favor formation of helical structures, and only a small fraction of β-structures is observed in long floating segments (Fig 3). Namely, *F(helix|long*, *flo)* = 0.664, and *F(beta|long*, *flo)* = 0.0410. This result reflects the fact that a floating, single α-helix is more stable than a floating single β-strand. A majority of long floating β-strands in the dataset have intermolecular contacts. A typical case is formation of an intermolecular β-sheet (Fig 4A; PDB ID: 2O8M). Floating helical segments are mainly observed at a solvated terminus of the chain (Fig 4B; PDB ID: 2WN9), in a coiled-coil (S2A Fig; PDB ID: 2GL2), and other types of interfaces (S2B Fig; PDB ID: 3RK0). For cases of unstructured (coil), long floating segments, typical cases are at the termini of proteins (S2C Fig; PDB ID: 3TER). The segments in the linker region are also observed (S2D Fig; PDB ID: 2B58). The tendency of longer segments to take helical structures is not observed in the supported segments, and the β-structures are not disfavored in long-supported segments (Fig 3). Since the usual size of β-sheets accords to the category of *T*_*len*_ = *middle*, a higher ratio of beta structures in the middle-supported segments are observed, compared with other lengths. Some examples of supported segments in each length are shown in Fig 4A, S3A and S3B Fig. Regarding coil structures, in contrast to the case of floating segments, only a small ratio of longer-supported segments adopts coiled structures. Typical long-supported segments with coil structures penetrate the domain (S3C Fig; PDB ID: 2BSL) or are located around the surface of the domain (S3D Fig; PDB ID: 3TEH).

### Segments as interfaces of intermolecular interactions

**Fig 5:**
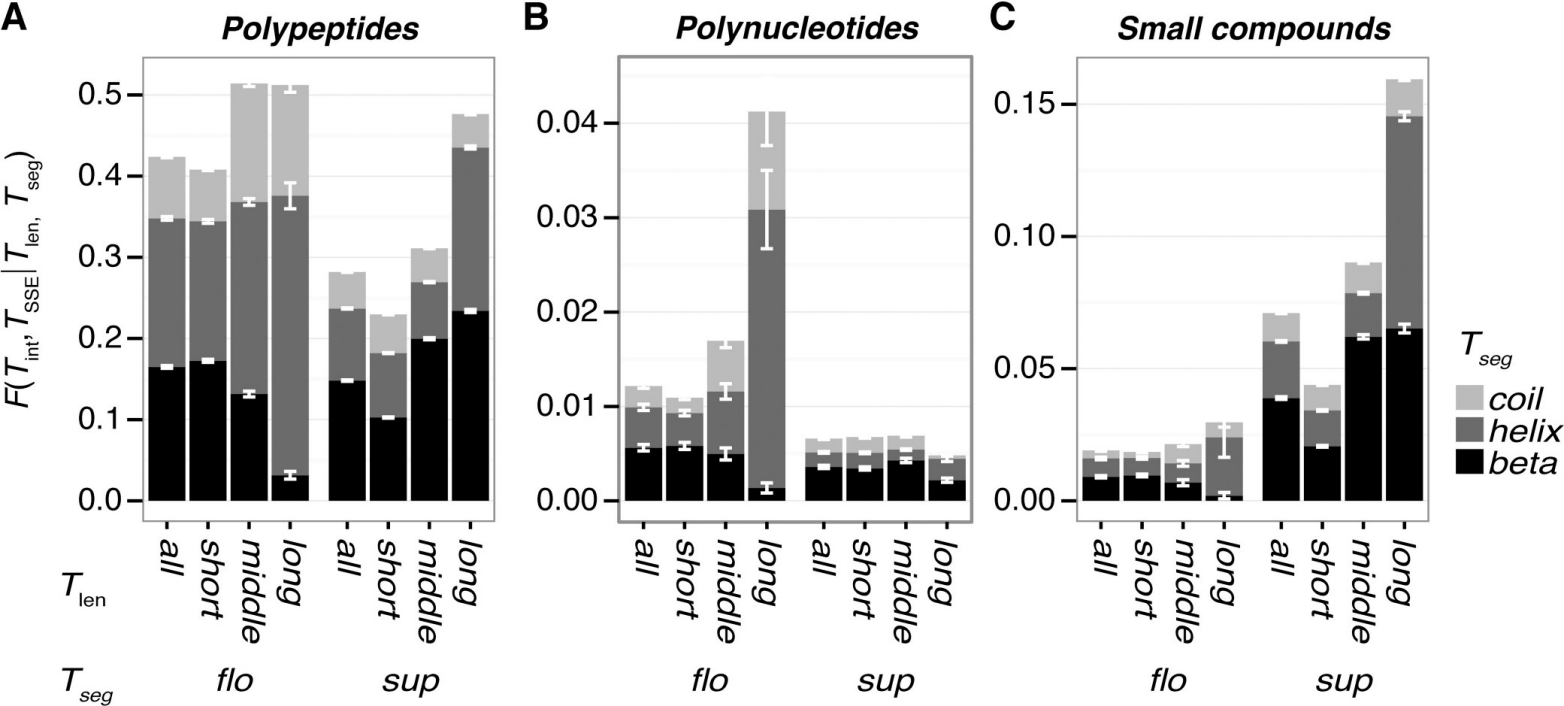
The ratios of interacting segments for each combination of classes: *T*_SSE_, *T*_len_ and *T*_seg_. (A) Ratios for interactions with polypeptides. (B) Ratios for interactions with polynucleotides. (C) Ratios for interactions with chemical compounds. Black, dark gray, and light gray denote the ratios of *beta*, *helix*, and *coil* structures, respectively. Error bars are the standard deviations in 100 non-redundant datasets.

The relative frequencies of segments for intermolecular interactions are summarized in Fig 5. The interaction partners were categorized into one of the three types, that is, polypeptides (*T*_*int*_ = *pep*), polynucleotides (*T*_*int*_ *= nuc*), or chemical compounds (*T*_*int*_ *= cc*). For interactions with polypeptides and polynucleotides, floating segments occur more frequently in intermolecular interfaces compared with supported segments; the ratio of floating segments that interact with polypeptides to all floating segments is *F(pep|flo)* = 0.424, and this ratio to all supported segments is *F(pep|sup)* = 0.282. In the case of polynucleotide interactions, these ratios to all floating segments and all supported segments are *F(nuc|flo)* = 0.0121 and *F(nuc|sup)* = 0.00659, respectively. This is because floating segments are on the surface of the subunit by definition. However, although floating segments are enriched at the surface, binding sites for chemical compounds prefer supported segments rather than floating ones; the ratios of interacting-floating and interacting-supported segments are *F(cc|flo)* = 0.0190 and *F(cc|sup)* = 0.0710, respectively. Since the binding sites are usually formed as a concave surface (called a cavity or pocket) with a certain size and depth [24], they should be formed by supported segments rather than by floating ones. As an example of binding sites with floating segments, 3-chlorocatechol 1,2-dioxygenase binds its ligand with a floating helix-turn-helix conformation (Fig 4D; PDB ID: 2BOY). In addition, many entries in this category do not have biologically relevant ligand-binding sites but have contacts with other non-specific chemical compounds such as lipids. For instance, a light harvesting complex is surrounded by chlorophyll molecules (S4 Fig; PDB ID: 3PL9).

Regarding the segment length, longer floating and supported segments show higher frequencies for interactions. This can be partially interpreted by the fact that longer segments have larger surface areas, which simply elevates the chances for interactions. For long-floating segments with helical structures, intermolecular interactions are typically formed by coiled-coil structures (S2A Fig). For long-floating segments with beta structures, intermolecular interactions are typically formed by intermolecular β-sheets (Fig 4A). As an exception to the tendency of the segment length, longer supported segments are not enriched in polynucleotide binding sites. Many typical double-stranded DNA binding sites include floating segments with positively charged amino acid residues. For example, bZIP heterodimeric complexes recognize DNA with two floating helices (Fig 4E; PDB ID: 2WT7). A floating-helix segment consisting of 18 residues in the NC2–TBP–DNA ternary complex structure recognizes the DNA with their six acidic residues (S5A Fig; PDB ID: 1JFI). A linker loop with two acidic residues in a replication terminator protein is buried into the major groove of DNA (S5B Fig; PDB ID: 1ECR). In contrast, it is sterically difficult to attach grooves of a double-stranded DNA to supported segments. A majority of supported segments at the polynucleotide binding sites touch the DNA backbone rather than burying into the grooves (for example PDB ID: 3E3Y; S5C Fig). In addition, many single-stranded DNA binding sites are composed of supported segments (for example S5D and S5E Fig; PDB ID: 2KFN and 3CMW, respectively).

### Propensities of amino acids

**Fig 6:**
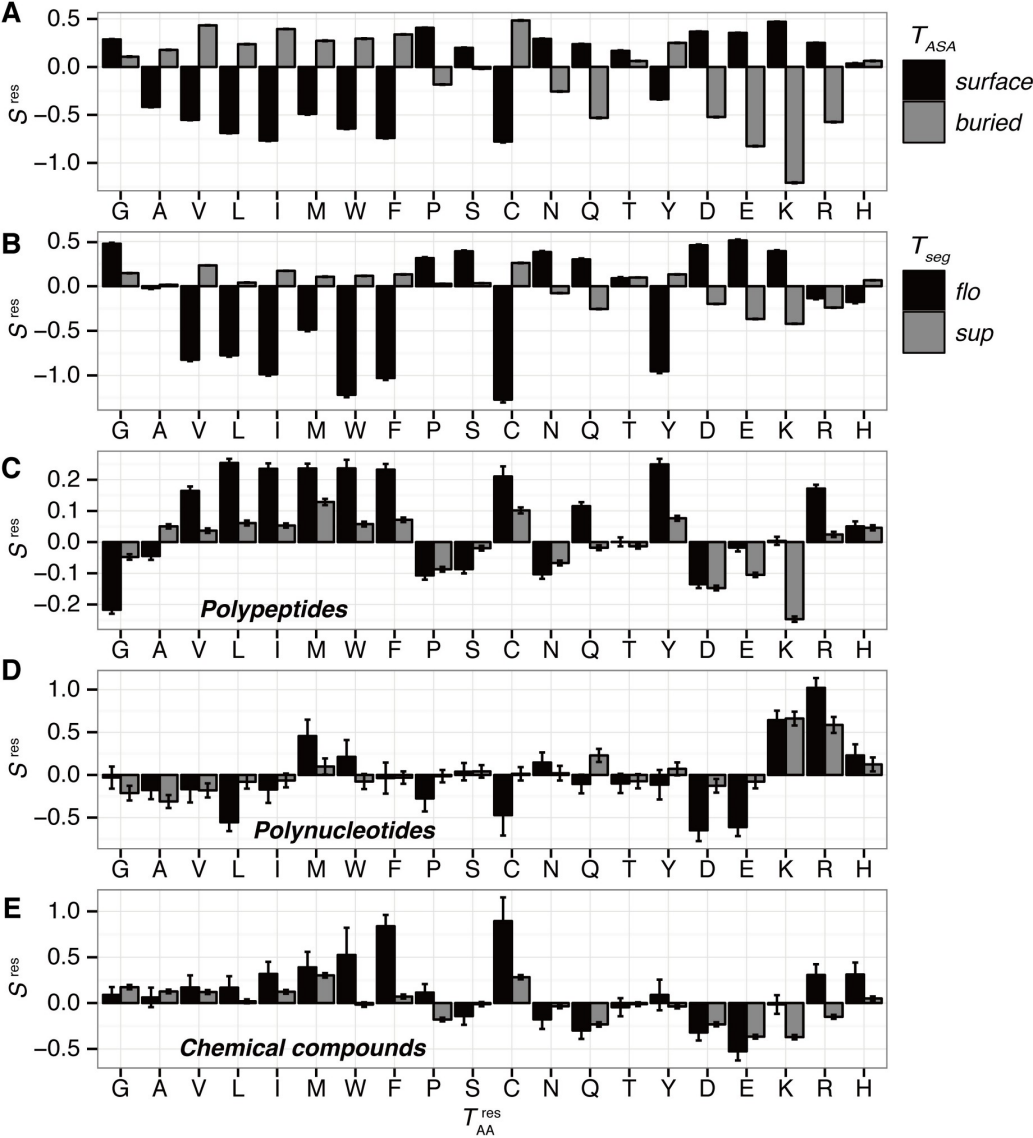
Amino acid propensity scores. (A) The propensities for surface or buried residues; $S^{res}(T_{AA}^{res}|T_{ASA})$. Black and gray bars denote surface and buried residues for the panel, respectively. (B) The propensities to form floating or supported segments; $S^{res}(T_{AA}^{res}|T_{seg})$. (C) The propensities to interact with polypeptides; $S^{res}(T_{AA}^{res}|T_{int}=pep;\;T_{seg})$. (D) The propensities to interact with polynucleotides; $S^{res}(T_{AA}^{res}|T_{int}=nuc;\;T_{seg})$. (E) The propensities to interact with chemical compounds; $S^{res}(T_{AA}^{res}|T_{int}=sc;\;T_{seg})$. Black and gray bars indicate floating and supported segments for the panels (B), (C), (D), and (E). Error bars are the standard deviations in 100 non-redundant datasets.

**Fig 7:**
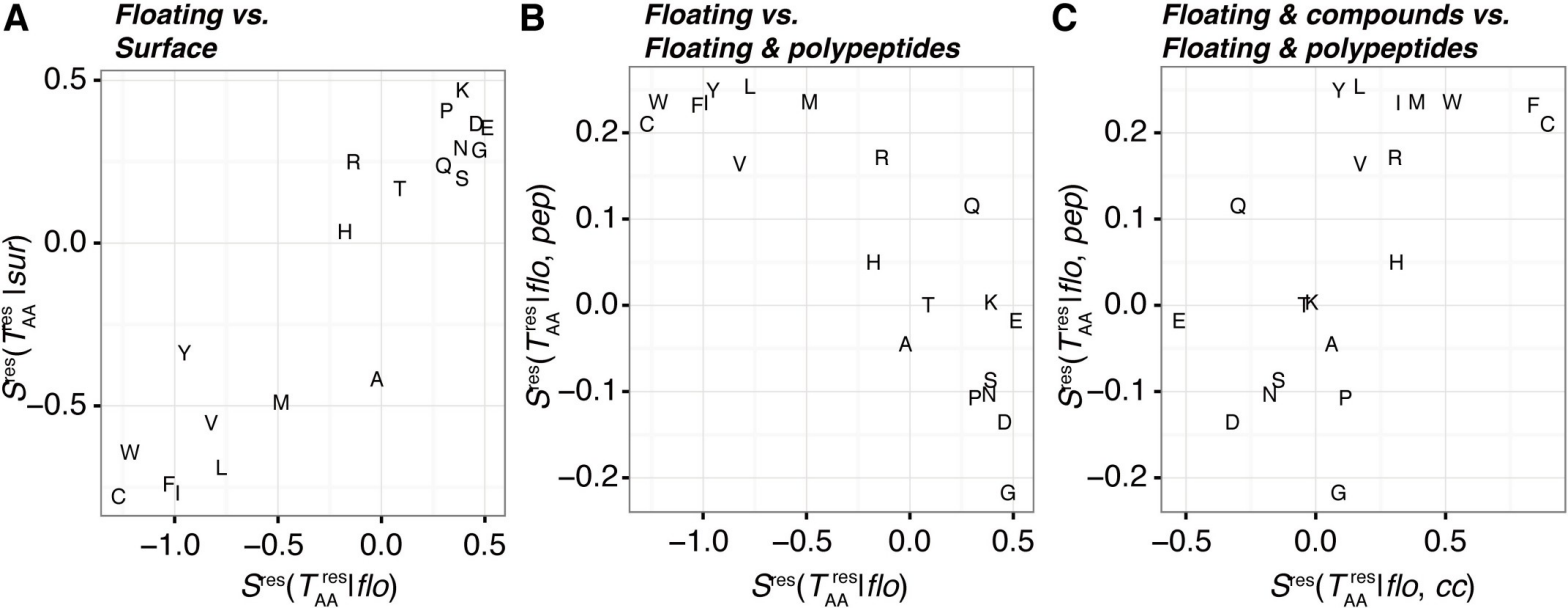
Comparisons of the amino acid propensity scores shown in Fig 6. (A) Comparison between floating and surface segments. Although the propensities of these types of segments are similar, floating segments show unique features. (B) Comparison between floating segments and those with peptide interactions. The opposite trend indicates that disfavored amino acids in floating segments are often interaction interfaces to other polypeptides. (C) Comparison between floating segments with chemical compound-interactions and those with peptide interactions. A weak correlation means that the formation of chemical compound-binding sites by floating segments has different trends from that of polypeptide-binding interfaces.

The propensity of each amino acid for floating and supported segments was assessed based on the log-odds score (Fig 6B; Eq 2). In general, bulky non-polar amino acids, e.g., Cys, Phe, Ile, Leu, Met, Val, Trp, and Tyr, are disfavored for floating segments. This tendency is similar for surface residues (Fig 7A; the Pearson correlation coefficient (PCC) of propensity scores between floating segments and surface residues is 0.929). This is due to the fact that a majority of floating segments are at the surface; *F(sur|flo)* = 0.953. However, there are some unique features in the amino acid propensity for the floating segments compared to surface residues. (i) Arg and His are disfavored in floating segments, although they are highly enriched as surface residues due to their high polarity. In many cases, they are involved in the interfaces of intermolecular contacts. For example, a 20-residue segment in the Fos-Jun complex has seven Arg residues, and six of them are in contact with DNA (Fig 4E; PDB ID: 1A02). It is well known that these residues are enriched for polynucleotide-binding interfaces [25,26]. Zhang et al. reported a review for structural bioinformatics studies and their analyses of amino acid propensities for interactions; this concluded that positively charged residues are favored for polynucleotide interactions [25]. Our results are roughly consistent with this (Fig 6D). At an interface with polypeptides, Arg can stabilize the interactions through the formation of salt bridges (S6A Fig; PDB ID: 2E7S). (ii) Gly is preferred for floating segments, although it is not so favored in surface residues. This is due to its high flexibility, which makes it possible to form unstructured regions, including loops and linkers. For example, a 12-residue segment in a loop region of MHC molecules has five Gly residues (S6B Fig; PDB ID: 1LNU). (iii) Ala is not so disfavored for the floating segments, in spite of its negative score in surface residues due to its hydrophobic side chain. One possible explanation is that α-helices favor Ala residues [10,27]. Floating segments show higher ratios of the helix conformation than supported segments (Fig 3). An example of a floating helix with many Ala residues is shown in S6C Fig (PDB ID: 4KE2).

We also assessed amino acid propensities for intermolecular interactions with the three categories of molecules: polypeptides, polynucleotides, and chemical compounds (Fig 6C, 6D and 6E, respectively). The propensity score of floating segments for polypeptide interactions (black in Fig 6C) shows the opposite trend from the propensity to form floating segments (Fig 7B; their PCC is -0.872). Although hydrophobic amino acids are disfavored for floating segments, they are favored for intermolecular interaction interfaces with other polypeptides. This implies that when disfavored amino acids exist in a floating segment, it is expected that they conduct some functions to recognize another protein. The exception is Gln, which has a positive propensity score for both conditions; *S*^*res*^*(Q; flo)* and *S*^*res*^*(Q*; *pep|flo)* shown in Fig 6B and 6C, respectively. Gln is often observed at terminal or kinking regions of a helix (examples are shown in S6D and S6E Fig). In addition, the direct comparison between intra- and intermolecular interactions indicates differences between them (S7 Fig). The propensity for floating segments in chemical compound-binding sites showed a weak correlation to that for polypeptides (Fig 7C; the PCC is 0.614). The major differences are as follows: Gly and Pro are favored, and Gln is disfavored for floating chemical compound-binding sites. Since Gly and Pro are enriched in flexible regions, they are observed in loop regions composing a binding site (examples are shown in S6F and S6G Fig). For the propensity for floating segments in interfaces to polynucleotide, there is no clear correlation with other propensities. In a comparison with the trend in supported segments, floating segments disfavor some hydrophobic amino acids, e.g., Cys, Leu, Pro, and Trp. In addition, while Asp and Glu are disfavored, Asn and Gln are not. They sometimes have direct contacts with a base of polynucleotides (S6H and S6I Fig).

## Conclusions

In this study, we defined floating and supported segments involved in the 3D structure of proteins (Fig 1) and characterized them on the basis of statistical analyses of the PDB. We found considerable numbers of floating segments in known protein structures (0.72% of residues are in floating segments). The frequency distribution of segment length shows exponential decay along with an increase in the segment length, in both floating and supported segments. The length distribution of floating segments is more biased toward shorter regions than that of supported segments, and most of the floating segments are composed of three or four residues (Fig 2). Three is the minimum length of a segment in the definition; the segment length largely impacts its characteristics. Shorter floating segments tend to form secondary structures (Fig 3). Longer floating segments are enriched in intermolecular interaction interfaces. In particular, beta structures are favored for long-floating segments at the interfaces (Fig 5). Although floating segments are enriched at interfaces for polypeptides and polynucleotides, they are disfavored at interfaces for chemical compounds (Fig 5). Regarding the amino acid composition, while floating segments are basically similar to surface exposed residues, they have some unique features: higher preferences for small side chains (Gly and Ala) and disfavoring some charged side chains (Arg and His) compared to surface residues (Fig 6A and 6B). Interestingly, the propensity scores for polypeptide interactions of floating segments are in an opposite trend from that for all floating segments (Fig 6B and 6C). Residues disfavored for floating residues tend to be interfaces for protein–protein interactions at floating segments, except for Gln residues.

## Supporting information

S1 Fig The number of PDB entries in each cluster. (TIF)

S2 Fig Examples of long floating segments. The target segment, the chain including the segment, and other chains are shown in red, green, and gray, respectively. (A) A floating-helix segment in a coiled-coil (adhesin FadA; PDB ID: 2GL2). (B) A floating-helix segment at an intermolecular interface (respiratory complex I; PDB ID: 3RKO). (C) A floating-coil segment at a terminus (the membrane domain of respiratory complex I; PDB ID: 3TER). (D) A floating-coil segment at a linker region (diamine acetyltransferase 1; PDB ID: 2B58).
(TIF)

S3 Fig Examples of supported segments. The target segment, the chain including the segment, and other chains are shown in red, green, and gray, respectively. (A) A supported-short segment in a β-sheet (kinase PhoQ catalytic domain; PDB ID: 3CGZ). (B) A supported-long segment in a β-sheet (a tRNA synthetase; PDB ID: 3TEG). (C) A supported-coil segment penetrating a globular domain (a dihydroorotate dehydrogenase A; PDB ID: 2BSL). (D) A supported-coil segment surrounding a globular domain (a tRNA synthetase; PDB ID: 3TEH).
(TIF)

S4 Fig An example of floating segments interacting with lipids (a chlorophyll binding protein; PDB ID: 3PL9). The target segment, the chain including the segment, and other chains are shown in red, green, and gray, respectively. The lipid is shown in yellow.
(TIF)

S5 Fig Examples of nucleic acid binding sites with floating segments. The target segment, the chain including the segment, and other chains are shown in red, green, and gray, respectively. (A) A floating-helix segment (the NC2–TBP–DNA ternary complex; PDB ID: 1JFI). (B) A coil-floating segment (a replication terminator protein; PDB ID: 1ECR). (C) A supported segment in the restriction enzyme HindII (PDB ID: 3E3Y). (D) A supported segment in the Klenow fragment of a DNA polymerase (PDB ID: 2KFN). (E) A supported segment in RecA (PDB ID: 3CMW).
(TIF)

S6 Fig Examples of floating segments with specific amino acids. The target amino acid residues, the segment including the residues, and the chain including the segment are shown in cyan, red, and green, respectively. The binding partners are shown in yellow. (A) A floating segment including Arg (the yeast Sec2p GEF domain; PDB ID: 2E7S). Arg forms a salt-bridge with Asp in the other chain. (B) A floating segment with Gly residues (an MHC molecule; PDB ID: 1LNU). (C) A floating segment with Ala residues (Type I hyperactive antifreeze protei; PDB ID: 4KE2). (D, E) Floating fragments with Gln residues interacting with the other polypeptide: (D) Huntingtin (PDB ID: 4FE8) and (E) enoyl reductase InhA (PDB ID: 4R9R). (F, G) Floating segments including Gly residues at the binding interface of the chemical compound: (F) HIV-1 protease (PDB ID: 1SH9), and (G) ecdysone receptor (PDB ID: 2R40). (H) A floating segment interacting with the RNA by Pro residues (virus capsid; PDB ID: 1DDL). (I) A floating segment interacting with the siRNA duplex by Asn residues (Piwi protein; PDB ID: 2GBB).
(TIF)

S7 Fig Amino acid propensities for comparison between inter- and intramolecular contacts. The black bars indicate the log-odds propensity scores for segments with intramolecular contacts (or supported segments) without intermolecular contacts. The gray bars indicate those for segments with intermolecular contacts without intramolecular contacts. The intra- and intermolecular contacts have different amino acid propensities.
(TIF)

S1 Data Segments are recorded in a .csv file. Each column indicates the PDB ID; the type of segment (supported or floating); the length of the segment; the length category (short, medium, or long); the secondary structural elements (helix, beta, or coil); the number of interacting molecules for polypeptides, polynucleotides, and chemical compound; and the sequence of the segment.
(GZ) Click here for additional data file.
