# Supplementary figures and images for: Characteristics of interactions at protein segments without non-local intramolecular contacts in the Protein Data Bank
Source: PLoS One. 2018 Dec 11;13(12):e0205052. doi: 10.1371/journal.pone.0205052 (PMC6289587; doi:10.1371/journal.pone.0205052)

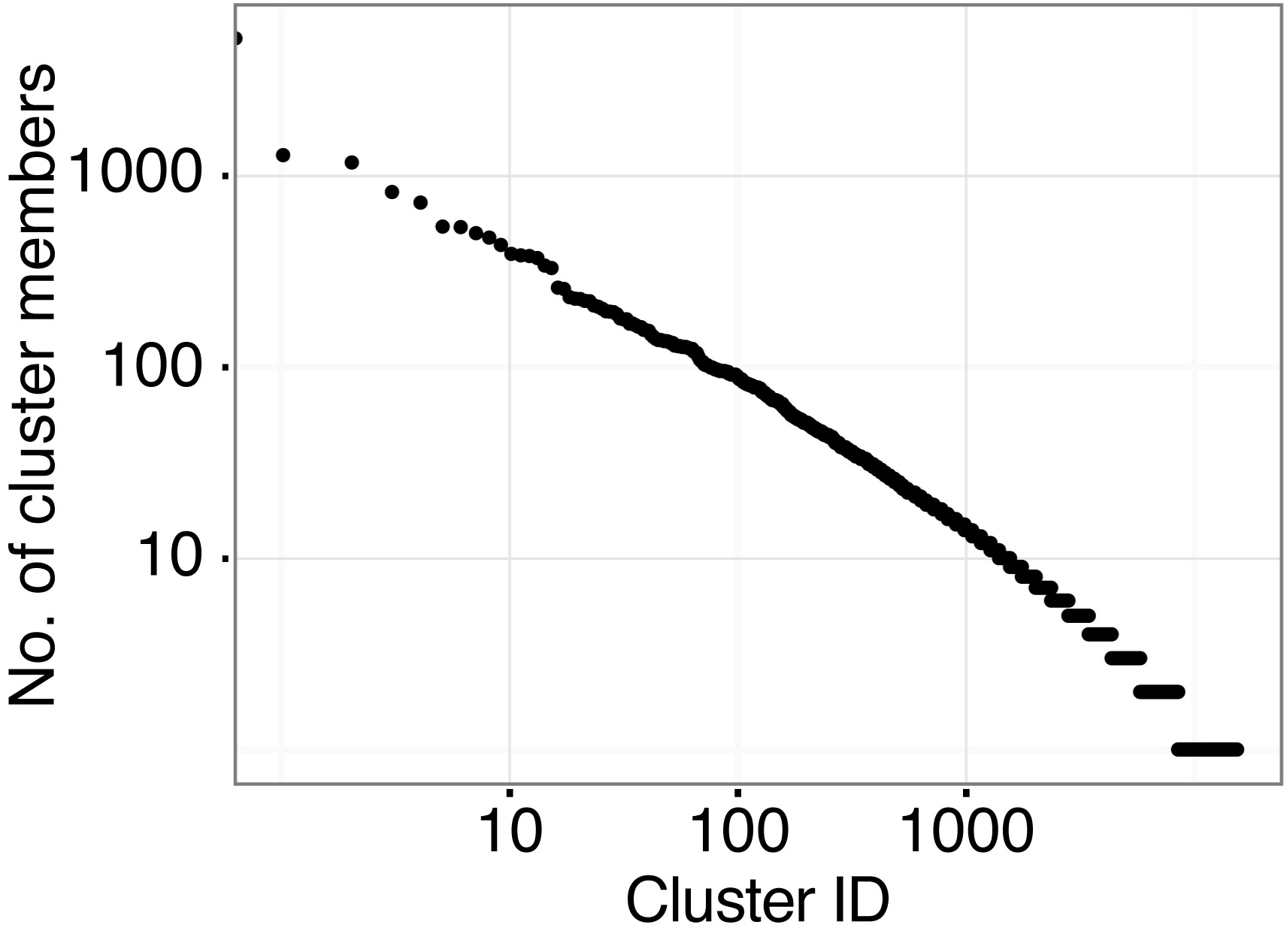

Supplement: S1 Fig — (TIF) [file pone.0205052.s001.tif]

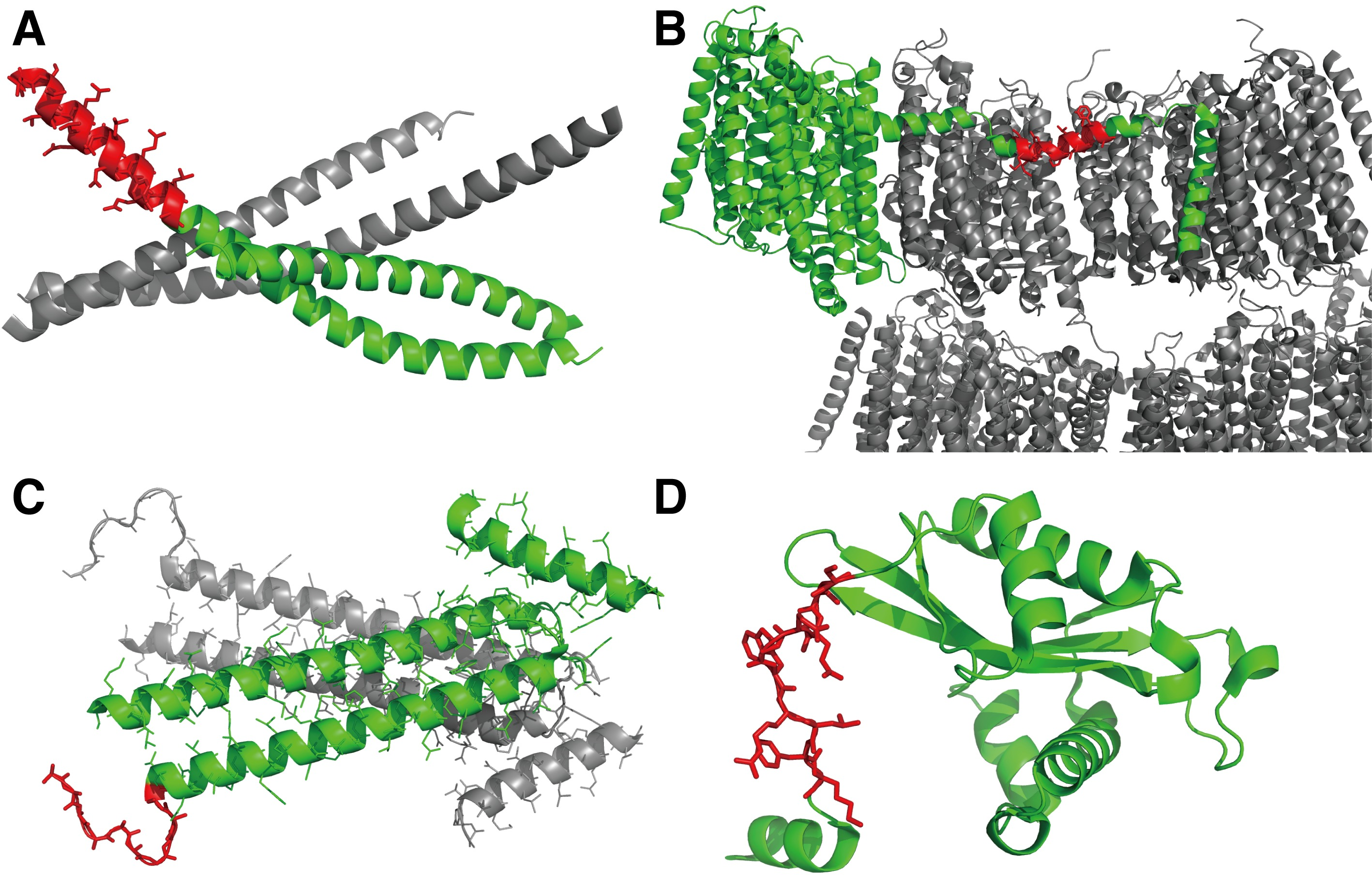

Supplement: S2 Fig — The target segment, the chain including the segment, and other chains are shown in red, green, and gray, respectively. (A) A floating-helix segment in a coiled-coil (adhesin FadA; PDB ID: 2GL2). (B) A floating-helix segment at an intermolecular interface (respiratory complex I; PDB ID: 3RKO). (C) A floating-coil segment at a terminus (the membrane domain of respiratory complex I; PDB ID: 3TER). (D) A floating-coil segment at a linker region (diamine acetyltransferase 1; PDB ID: 2B58). (TIF) [file pone.0205052.s002.tif]

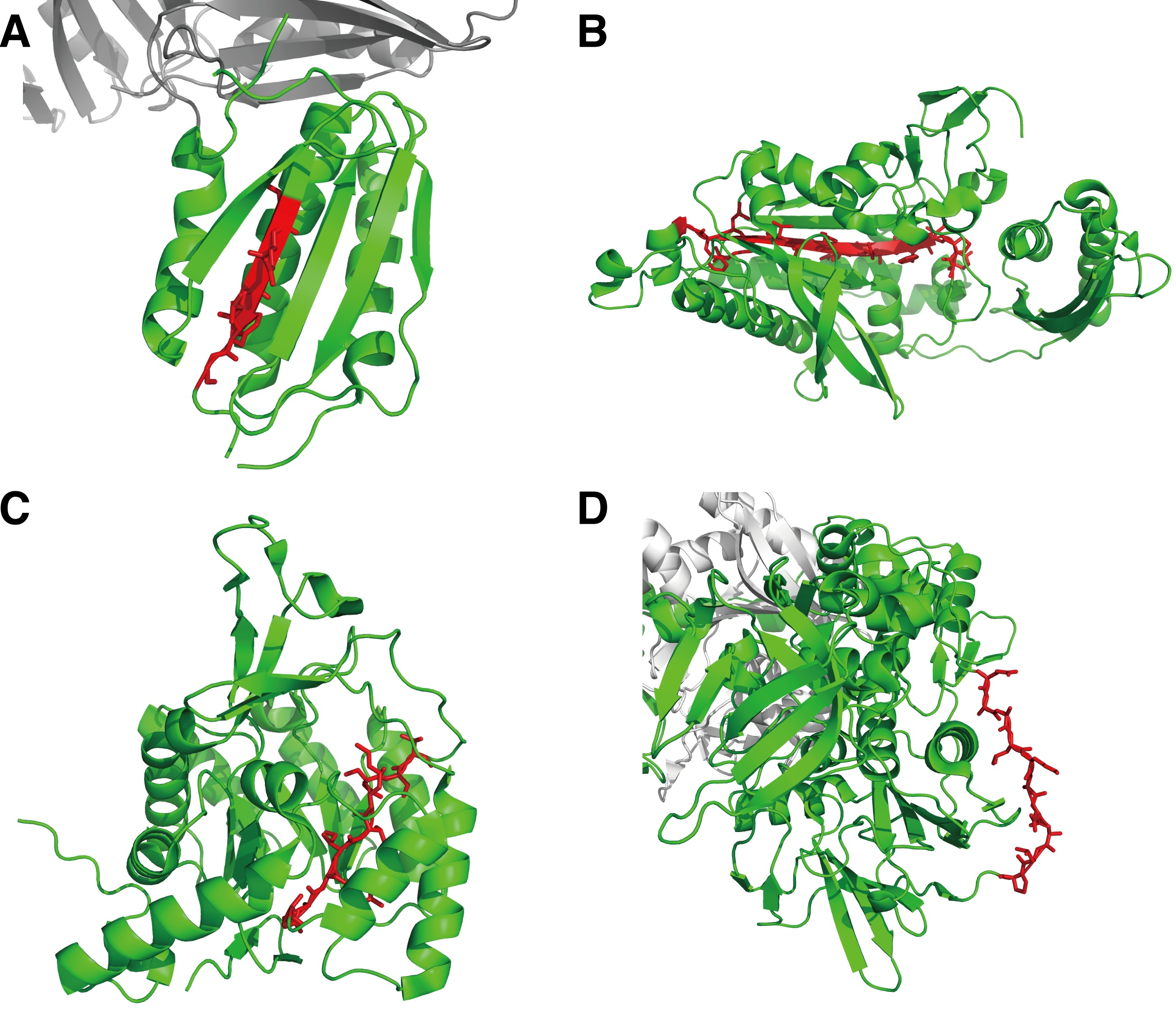

Supplement: S3 Fig — The target segment, the chain including the segment, and other chains are shown in red, green, and gray, respectively. (A) A supported-short segment in a β-sheet (kinase PhoQ catalytic domain; PDB ID: 3CGZ). (B) A supported-long segment in a β-sheet (a tRNA synthetase; PDB ID: 3TEG). (C) A supported-coil segment penetrating a globular domain (a dihydroorotate dehydrogenase A; PDB ID: 2BSL). (D) A supported-coil segment surrounding a globular domain (a tRNA synthetase; PDB ID: 3TEH). (TIF) [file pone.0205052.s003.tif]

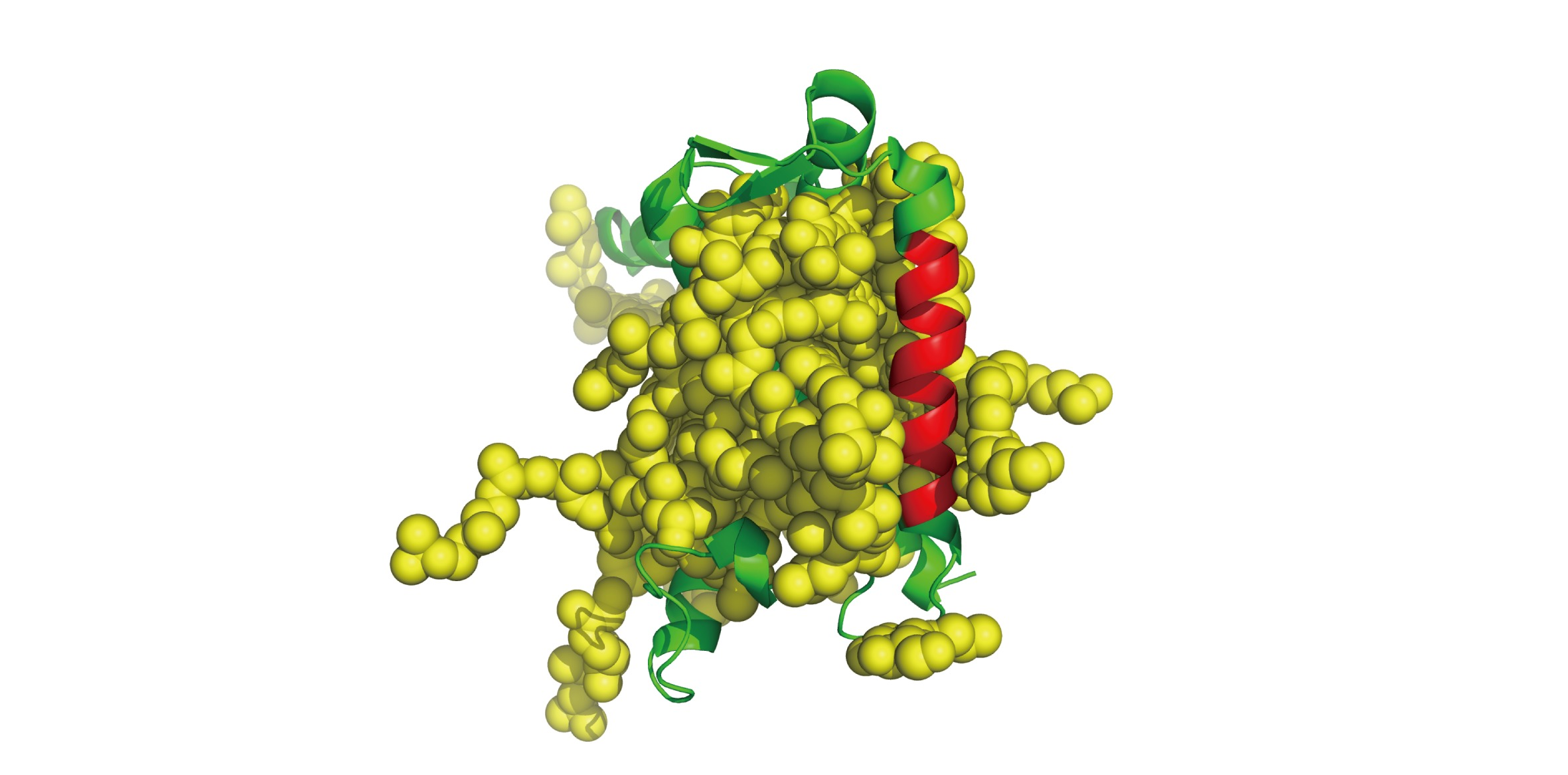

Supplement: S4 Fig — The target segment, the chain including the segment, and other chains are shown in red, green, and gray, respectively. The lipid is shown in yellow. (TIF) [file pone.0205052.s004.tif]

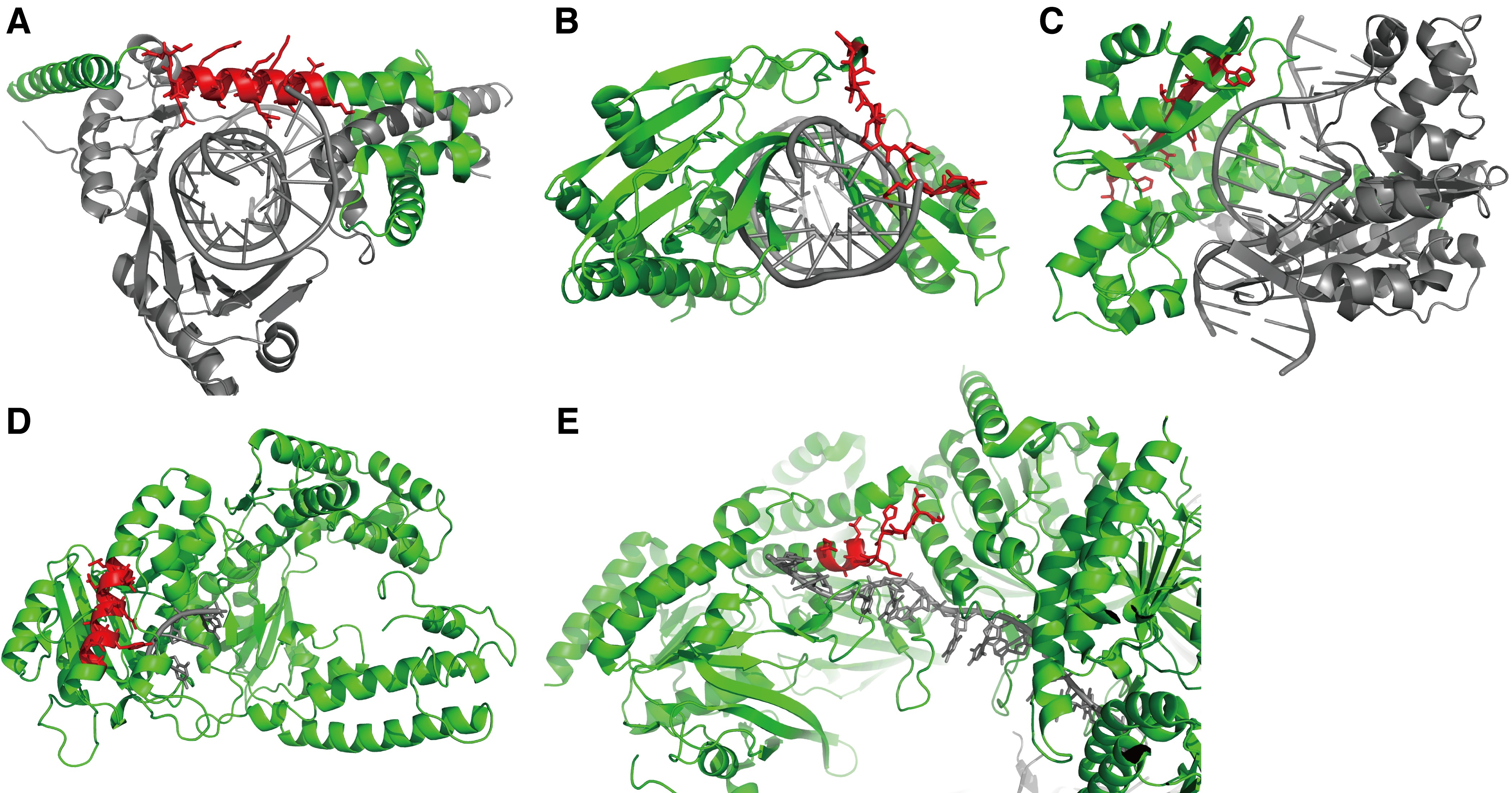

Supplement: S5 Fig — The target segment, the chain including the segment, and other chains are shown in red, green, and gray, respectively. (A) A floating-helix segment (the NC2–TBP–DNA ternary complex; PDB ID: 1JFI). (B) A coil-floating segment (a replication terminator protein; PDB ID: 1ECR). (C) A supported segment in the restriction enzyme HindII (PDB ID: 3E3Y). (D) A supported segment in the Klenow fragment of a DNA polymerase (PDB ID: 2KFN). (E) A supported segment in RecA (PDB ID: 3CMW). (TIF) [file pone.0205052.s005.tif]

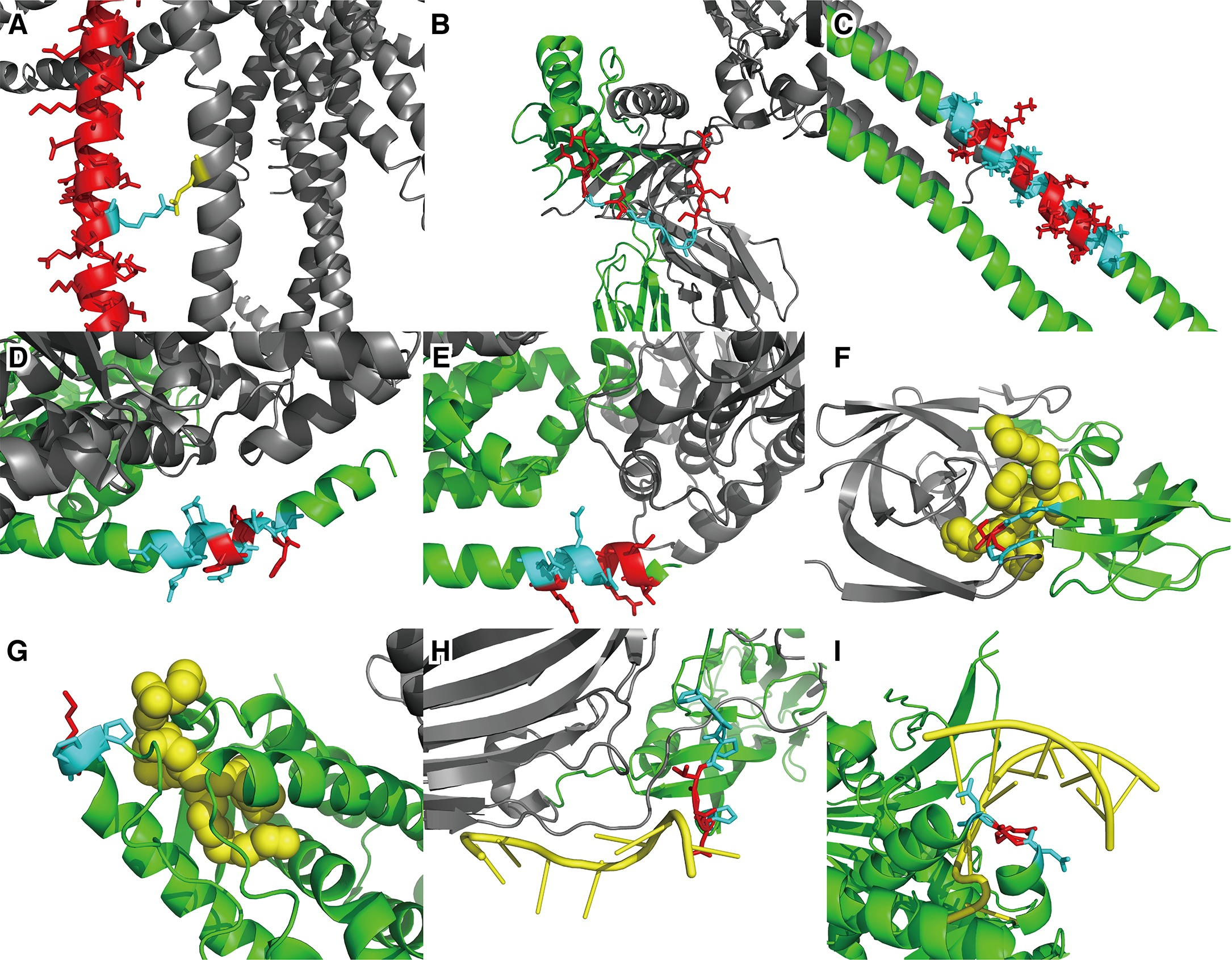

Supplement: S6 Fig — The target amino acid residues, the segment including the residues, and the chain including the segment are shown in cyan, red, and green, respectively. The binding partners are shown in yellow. (A) A floating segment including Arg (the yeast Sec2p GEF domain; PDB ID: 2E7S). Arg forms a salt-bridge with Asp in the other chain. (B) A floating segment with Gly residues (an MHC molecule; PDB ID: 1LNU). (C) A floating segment with Ala residues (Type I hyperactive antifreeze protei; PDB ID: 4KE2). (D, E) Floating fragments with Gln residues interacting with the other polypeptide: (D) Huntingtin (PDB ID: 4FE8) and (E) enoyl reductase InhA (PDB ID: 4R9R). (F, G) Floating segments including Gly residues at the binding interface of the chemical compound: (F) HIV-1 protease (PDB ID: 1SH9), and (G) ecdysone receptor (PDB ID: 2R40). (H) A floating segment interacting with the RNA by Pro residues (virus capsid; PDB ID: 1DDL). (I) A floating segment interacting with the siRNA duplex by Asn residues (Piwi protein; PDB ID: 2GBB). (TIF) [file pone.0205052.s006.tif]

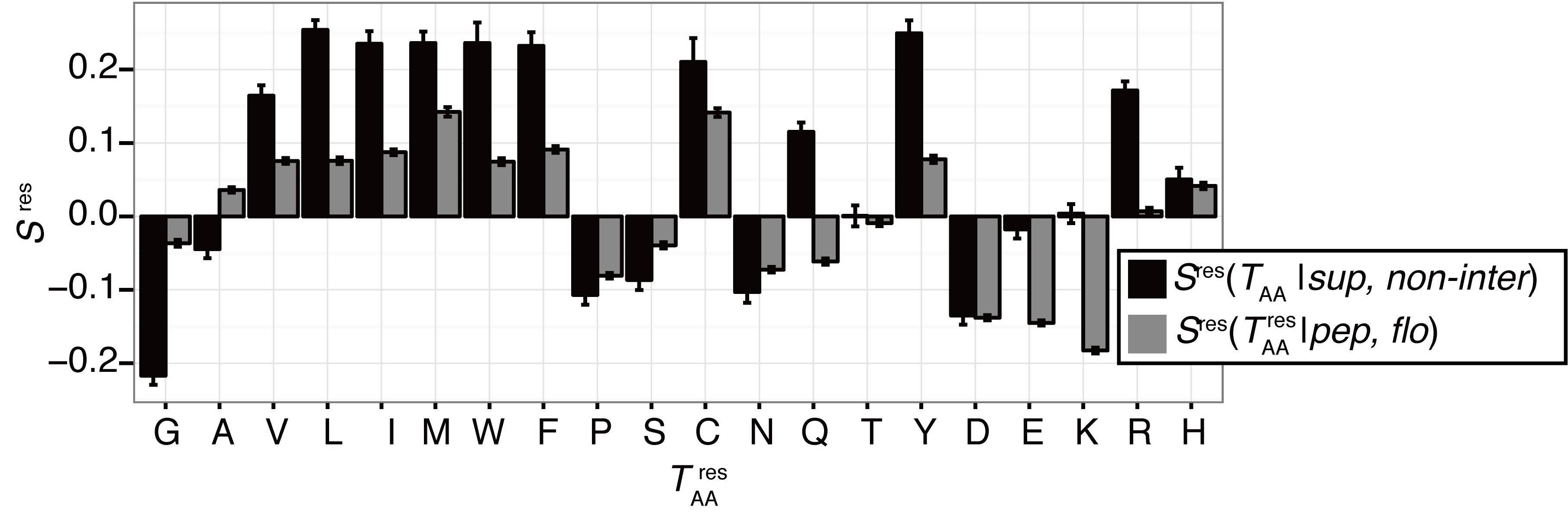

Supplement: S7 Fig — The black bars indicate the log-odds propensity scores for segments with intramolecular contacts (or supported segments) without intermolecular contacts. The gray bars indicate those for segments with intermolecular contacts without intramolecular contacts. The intra- and intermolecular contacts have different amino acid propensities. (TIF) [file pone.0205052.s007.tif]
